# Diversity and regulation of ATP sulfurylase in photosynthetic organisms

**DOI:** 10.3389/fpls.2014.00597

**Published:** 2014-11-05

**Authors:** Laura Prioretti, Brigitte Gontero, Ruediger Hell, Mario Giordano

**Affiliations:** ^1^Laboratory of Algal and Plant Physiology, Dipartimento di Scienze della Vita e dell'Ambiente, Università Politecnica delle MarcheAncona, Italy; ^2^Aix-Marseille Université Centre National de la Recherche Scientifique, BL' Unité de Bioénergétique et Ingénierie des Protéines UMR 7281Marseille, France; ^3^Centre for Organismal Studies, University of HeidelbergHeidelberg, Germany; ^4^Institute of Microbiology Academy of Sciences of the Czech RepublicTrebon, Czech Republic

**Keywords:** algae, algal evolution, ATPS, cysteine, redox regulation, sulfur metabolism

## Abstract

ATP sulfurylase (ATPS) catalyzes the first committed step in the sulfate assimilation pathway, the activation of sulfate prior to its reduction. ATPS has been studied in only a few model organisms and even in these cases to a much smaller extent than the sulfate reduction and cysteine synthesis enzymes. This is possibly because the latter were considered of greater regulatory importance for sulfate assimilation. Recent evidences (reported in this paper) challenge this view and suggest that ATPS may have a crucial regulatory role in sulfate assimilation, at least in algae. In the ensuing text, we summarize the current knowledge on ATPS, with special attention to the processes that control its activity and gene(s) expression in algae. Special attention is given to algae ATPS proteins. The focus on algae is the consequence of the fact that a comprehensive investigation of ATPS revealed that the algal enzymes, especially those that are most likely involved in the pathway of sulfate reduction to cysteine, possess features that are not present in other organisms. Remarkably, algal ATPS proteins show a great diversity of isoforms and a high content of cysteine residues, whose positions are often conserved. According to the occurrence of cysteine residues, the ATPS of eukaryotic algae is closer to that of marine cyanobacteria of the genera *Synechococcus* and *Prochlorococcus* and is more distant from that of freshwater cyanobacteria. These characteristics might have evolved in parallel with the radiation of algae in the oceans and the increase of sulfate concentration in seawater.

## Introduction

Sulfur is an element of primary importance for all living organisms because it is a component of a very large number of compounds with essential biological functions (Giordano and Prioretti, 2014; Giordano and Raven, 2014; Glaeser et al., 2014). Photosynthetic organisms acquire S at its highest oxidation number (as sulfate), but assimilate it in its most reduced form of sulfide. The assimilation into organic molecules occurs primarily through cysteine from which S is then redistributed to the other sulfur amino acid, methionine, and other S-containing compounds (Takahashi et al., 2011; Giordano and Prioretti, 2014; Giordano and Raven, 2014). Due to the high reactivity of thiol (–SH) groups, S-compounds such as cysteine and glutathione are pivotal for metabolic redox regulation (Couturier et al., 2013). Sulfur acquisition and assimilation have been thoroughly described for vascular plants (Takahashi et al., 2011) and appear to be mostly conserved in the other photoautotrophic organisms, although differences exist in parts of the pathway (Giordano and Prioretti, 2014; Giordano and Raven, 2014).

ATP sulfurylase (ATPS; ATP:sulfate adenylyltransferase, EC 2.7.7.4) is the first enzyme of the sulfate assimilation pathway (Takahashi et al., 2011). Although a fair amount of information is available for fungal and bacterial ATPS (Mueller and Shafqat, 2013), the ATPS of phototrophic organisms (especially of algae) has not been studied as much as the enzymes that catalyze sulfate reduction (adenosine 5′-phosphosulfate reductase, APR) and cysteine synthesis (cysteine synthase or O-acetylserine (thiol)lyase, OAS-TL). This is probably the consequence of the fact that sulfate assimilation, in vascular plants, is mostly regulated through these enzymes (Takahashi et al., 2011). New evidence (see below) however challenges this view and attributes a more important regulatory role to ATPS than previously believed. We attempted to put the structural and catalytic information on ATPS in an evolutionary context, in order to provide clues, although not yet definitive answers, on what selective processes led to the ATPS proteins present in the extant organisms.

## ATP sulfurylase reaction and catalytic mechanism

**Figure 1:**
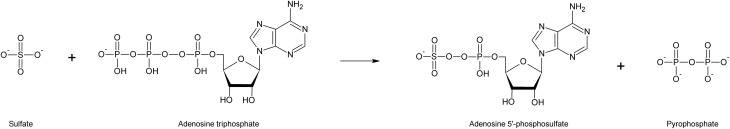
**Reaction catalyzed by ATP sulfurylase**. The enzyme performs the hydrolysis of the bond between the α- and β-phosphate of ATP and, consequently, the attachment of AMP to SO^2−^_4_. MgPPi is finally released.

**Figure 2:**
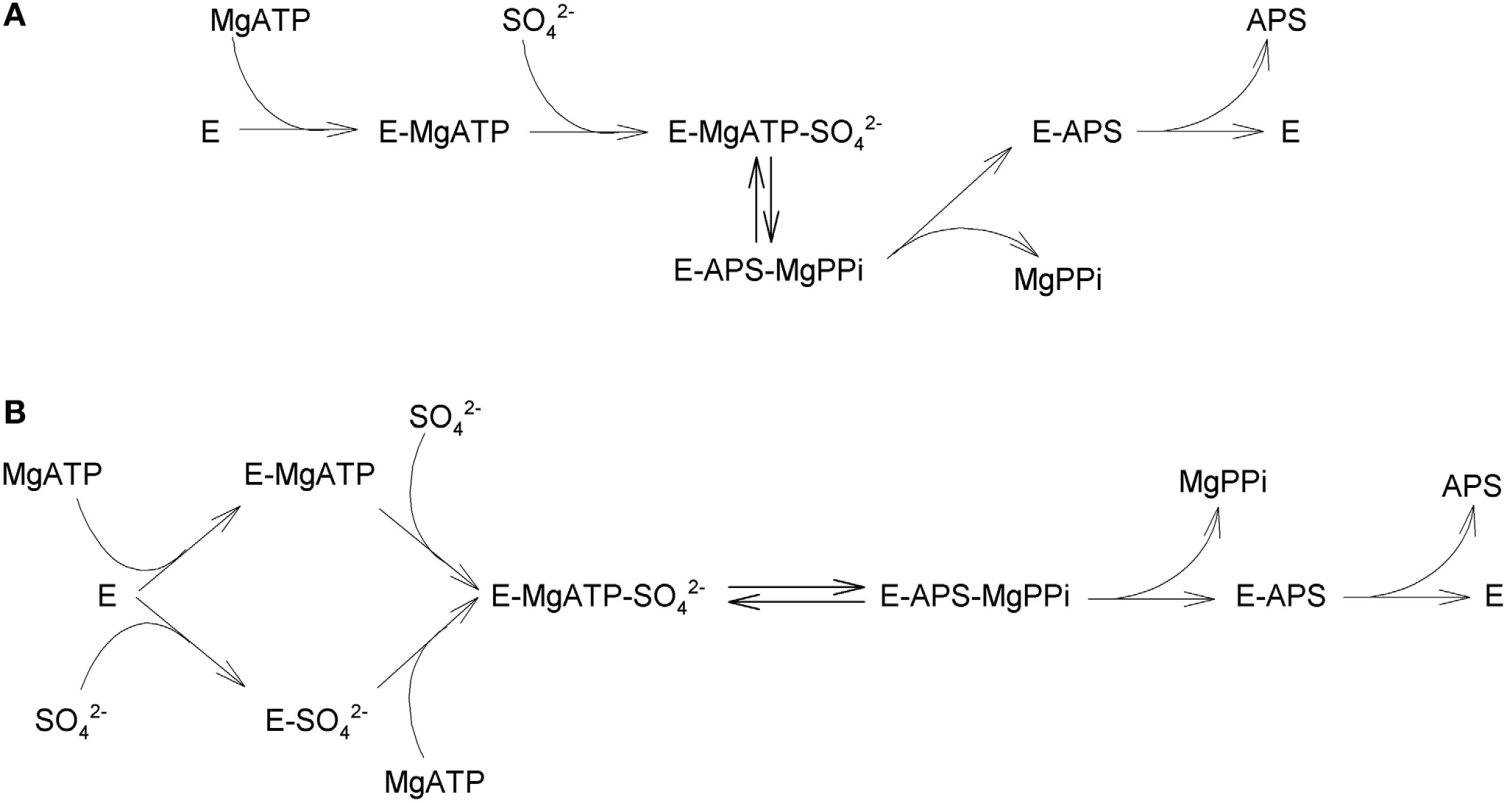
**ATP sulfurylase reaction mechanism**. Panel **(A)** depicts the ordered reaction mechanism described by Farley et al. (1976) and Ravilious et al. (2013); panel **(B)** depicts the random reaction sequence as described by Seubert et al. (1983, 1985).

ATPS is a nucleotidyl transferase that belongs to the superfamily of α/β phosphodiesterases. It catalyzes the non-reductive adenylation of sulfate to adenosine 5′-phosphosulfate (APS) and pyrophosphate (PPi) (Bicknell et al., 1982; Figure 1). Early studies on fungi (Farley et al., 1976, 1978) and plant (Shaw and Anderson, 1974) suggested that ATPS catalyzes an ordered reaction, in which MgATP is the first substrate to bind the enzyme active site (E); sulfate (SO^2−^_4_) would then interact with the E-MgATP complex and ATP is cleaved. Finally AMP is bound to sulfate, with a concomitant release of MgPPi; as the final step, APS is released from the active site (Figure 2A).

Later studies, however, disputed this mechanism and proposed that, at least in *Penicillium*, the entrance of ATP and SO^2−^_4_ in the active site can occur in a random order (Seubert et al., 1983). The main evidence supporting the random catalytic model is that APS, the final product of ATPS catalysis, strongly competes with the binding of both MgATP and SO^2−^_4_ to the enzyme (Seubert et al., 1983). Seubert et al. (1985) also showed that all kinetic data are consistent with a random substrate binding mechanism for the forward reaction of ATPS, whereas the reverse reaction follows an ordered mechanism in which MgPPi is the first substrate to bind and leave the ATPS active site (Figure 2B). A recent report suggested an ordered mechanism, with sulfate binding after ATP, in the ATPS from *Glycine max*. For the reverse reaction, kinetic analysis and isothermal titration calorimetry-binding studies for an *Arabidopsis thaliana* ATPS isoform indicate that APS binding occurs first, followed by addition of PPi (Ravilious et al., 2013), in contrast to what was reported for the fungal enzyme (Seubert et al., 1985). The investigation of the catalytic mechanisms and the resolution of three-dimensional structure of more ATPS proteins may lead to the clarification of these open questions.

## Sulfate activation

The incorporation of sulfate (SO^2−^_4_) into organic molecules requires the reduction of sulfate to sulfite (SO^−^_3_). The standard potential, *E*_0_', of this redox pair, however, is extremely negative (sulfate/sulfite *E*_0_' = −454 mV, pH 7 and 25°C), and this reaction is thermodynamically impossible in a biological context because there are no reductant carriers in the cell that have a sufficiently negative redox potential. Sulfate, therefore, needs to be activated to APS by ATPS. APS can in fact be reduced to sulfite at a much lower redox potential, since the *E*_0_' for the pair APS/sulfite is of only −60 mV, well within the range of redox potential of the biological carriers of reducing power (Rauen, 1964; Segel, 1976).

Sulfate activation is energetically aided by the subsequent cleavage of the phosphoric-sulfuric acid anhydride bond of APS for the production of sulfite, with the catalysis of APS reductase (APR). Such reaction has a ΔG^0^ of about −19 kcal mol^−1^ (Segel, 1976), a rather high value compared, for example, to the energy generated by the hydrolysis of the phosphate-phosphate bond of an ATP (ΔG^0^ approximately −8 kcal mol^−1^; Schiff and Hodson, 1973). The degradation of APS is also important because of the strong product inhibition of ATPS (Farley et al., 1976). It is thus not surprising that the enzymes that operate downstream of ATPS, i.e., APR and APS kinase (APK) (see Giordano and Prioretti, 2014 for details on the metabolic pathways downstream of ATPS) have a high affinity for APS [*K*_*m*(APS)_ = 2.1 μmol L^−1^ for the red macroalga *Porphyra yezoensis* APR; (Bick and Leustek, 1998); *K*_*m*(APS)_ = 1–10 μmol L^−1^ for *A. thaliana* APK; Lee and Leustek, 1998; Lillig et al., 2001]. *In vivo*, the activity of pyrophosphatases is believed to contribute to pull the reaction toward the production of APS, then facilitating the overall process of sulfate activation (ΔG^0^ pyrophosphatase = −5 kcal mol^−1^; Bicknell et al., 1982).

The fate of APS has been the subject of scholarly disputes for a rather long time. It was proposed that ATPS and APK worked together in a channeling mechanism that would allow the direct phosphorylation of APS to PAPS (Seubert et al., 1983; Sun and Leyh, 2006); this hypothesis was especially attractive because it could explain the existence of coupled ATPS-APK (PAPS synthetase) in metazoa (Mueller and Shafqat, 2013), fungi (MacRae et al., 2002), oomycetes and some algae (Patron et al., 2008). Experiments were carried out on enzymes from a variety of organisms to verify this hypothesis, but, so far, the channeling mechanism was demonstrated only for the purple bacterium *Rhodobacter sphaeroides* (Sun and Leyh, 2006). The existence of a channeling mechanism between ATPS and APR was also hypothesized for the *in vitro* formation of an ATPS-APR complex in *Allium cepa* (Cumming et al., 2007), but the occurrence of this complex *in vivo* and its functional significance are still unclear.

In plants and, as far as we know, in algae, the APS produced by ATPS is used (i) by APR for the production of sulfite in the chloroplast (only the euglenozoan *Euglena gracilis* seems to reduce sulfate in the mitochondrion; Brunold and Schiff, 1976) and (ii) by APK in plastid and cytosol for the production of PAPS, which is the main substrate for sulfation reactions (Takahashi et al., 2011; Giordano and Prioretti, 2014).

## ATPS isoforms

ATPS is present in both photosynthetic and non-photosynthetic organisms, in both prokaryotes and eukaryotes, and is involved in a variety of S-dependent processes. In proteobacteria, the genes for sulfate uptake and assimilation are organized in the *cys* operon, which in *Escherichia coli* and *Salmonella typhimurium* is composed of 18 genes (Leyh, 1993). Two of these genes, *cysD* and *cysN*, encode, respectively, the small (27 kDa) and the large (62 kDa) subunit of the heterodimeric ATPS (Leyh et al., 1988). The main peculiarity of bacterial ATPS is that the cysN subunit possesses a GTP-binding site and acts as a GTPase (Liu et al., 1994; Mougous et al., 2006). The energy derived from the hydrolysis of GTP fuels the ATPS activity of cysD (Leyh, 1993; Liu et al., 1994). Surprisingly, in the symbiotic diazotrophic bacterium *Rhizobium meliloti*, genes analogous to *cysD* and *cysN*, named *nodP* and *nodQ*, were found in the cluster of the nodulin genes where they participate to the biosynthesis of a sulfated nodulation factor (Schwedock and Long, 1990).

**Figure 3:**
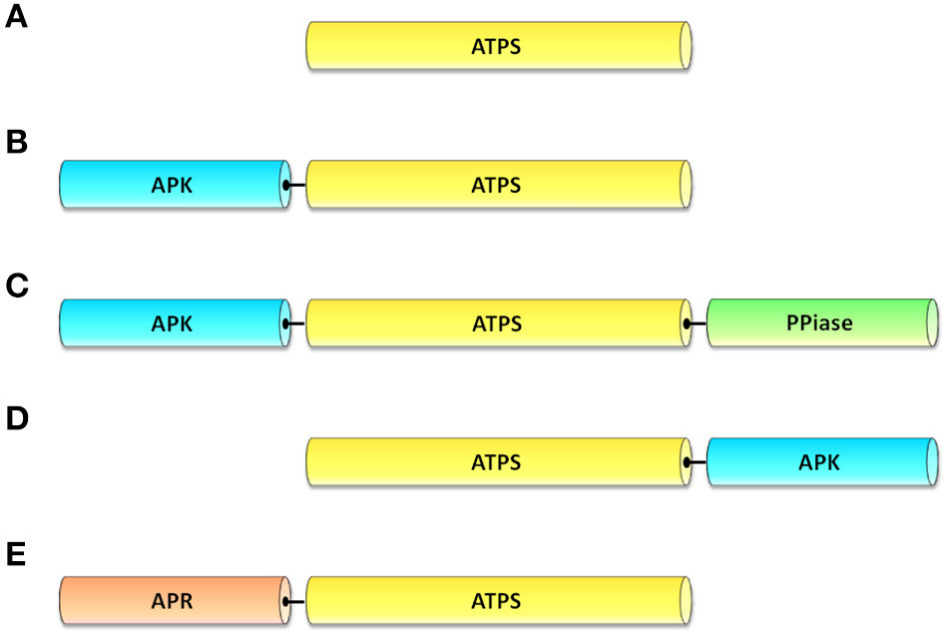
**Domain models of ATP sulfurylase (ATPS). (A)** ATPS proteins of cyanobacteria, green algae, red algae, plastidial isoform of “red lineage” algae (with the exception of *T. pseudonana*). **(B)** metazoan PAPS synthetase with an APS kinase (APK) domain at the N-terminus of the ATPS domain. **(C)** cytosolic ATPS proteins of diatoms and haptophytes with an APK domain at the N-terminus and a pyrophosphatase (PPiase) domain at the C-terminus. **(D)** ATPS proteins of fungi and of the thermophylic bacterium *Aquifex aeolicus* fused with an APK domain at the C-terminus. **(E)** ATPS proteins of dinoflagellates, the apicomplexan *Chromera velia* and the filasterian *Capsaspora owczarzaki*, fused with an APS reductase (APR) at the N-terminus.

The crystalline structure of the thermophile *Thermus thermophilus* (Taguchi et al., 2004), the purple sulfur bacterium *Allochromatium vinosum* (Parey et al., 2013) and a symbiont of the hydrothermal vent tubeworm *Riftia pachyptila* (Beynon et al., 2001) revealed that, in these organisms, ATPS is a homodimeric enzyme. Also the ATPS crystal structure of the hyperthermophile bacterium *Aquifex aeolicus* shows that the enzyme is homodimeric and, differently from the above mentioned species, contains a functional APK domain at the ATPS C-terminus (Yu et al., 2007). Similarly to *A. aeolicus*, fungi possess an ATPS fused to an APK-like motif at the C-terminus (Figure 3D). In *Penicillium chrysogenum*, the two domains are organized in a homohexamer of 63.7 kDa subunits (see crystal structure in MacRae et al., 2001) and the APK domain carries out a PAPS-mediated allosteric regulation on the ATPS domain (MacRae et al., 2002). Also the ATPS of *Saccharomyces cerevisiae* is a homohexamer with 58 kDa monomers, as it emerges from the enzyme crystal structure (Ullrich et al., 2001); in this enzyme, however, the APK domain does not appear to have a catalytic function and probably has a structural role in the association of the ATPS monomers (Lalor et al., 2003).

Photosynthetic organisms contain a variable number of ATPS isoforms, with various degrees of sequence similarities. All vascular plants possess at least two ATPS isoforms, with the exception of the lycophyte *Selaginella moellendorffii* that only has one (Kopriva et al., 2009). In *A. thaliana*, an ATPS gene family of four members exists (*ATPS1-4*; Leustek et al., 1994; Murillo and Leustek, 1995; Hatzfeld et al., 2000). The four genes are located on different chromosomes. Although all four ATPS isoforms possess a plastidial transit peptide, there are indications that one of them is also expressed in the cytosol (Rotte and Leustek, 2000). Recently, the first plant ATPS crystal structure was obtained for *Glycine max* (Herrmann et al., 2014). The *Glycine max* ATPS is a homodimer of approximately 100 kDa, formed by two ~48 kDa monomers (Phartiyal et al., 2006; Ravilious et al., 2013). Surprisingly, sequence comparison and biochemical analyses revealed that plant ATPS is rather similar to the human enzyme (Patron et al., 2008), which, like soybean ATPS, is a homodimer (Harjes et al., 2005). Yet, the plant ATPS is a monofunctional enzyme (Figure 3A), whereas the human one is a bifunctional PAPS synthetase, with an APK domain fused through a linker at the ATPS N-terminus (Harjes et al., 2005; Figure 3B).

A very large degree of diversity has been found among algal ATPS proteins. The “green lineage” algae (those derived from the primary endosymbiotic event leading to Chl a+b algae; e.g., green algae) possess one plastidial ATPS isoform encoded by a single gene (Giordano and Prioretti, 2014; Figure 3A). The sole known exception is the freshwater green microalga *Chlamydomonas reinhardtii*, which possesses two ATPS proteins located in the plastid, which are encoded by two distinct nuclear genes, termed *ATS1* and *ATS2* (Patron et al., 2008). A larger degree of heterogeneity exists among the algae of the “red lineage” (those derived from the endosymbiosis leading to Chl a+c algae, e.g., red algae, diatoms, dinoflagellates and haptophytes). The few red algae for which an ATPS sequence is known possess one (*Galdieria sulphuraria*, *Pyropia yezoensis*, *Porphyra purpurea*) or two (*Cyanidioschyzon merolae* and *Chondrus crispus*) ATPS isoforms: *P. yezoensis* and *P. purpurea* ATPS enzymes are cytosolic; in *C. merolae* one isoform is plastidial and one is cytosolic (Patron et al., 2008); for the other species the location of ATPS is still unclear.

Diatoms, dinoflagellates and haptophytes possess two ATPS isoforms, one in the chloroplast, the other most likely in the cytosol (Patron et al., 2008). The plastidial enzyme is involved in primary sulfate assimilation. The cytosolic isoform of diatoms and haptophytes is presumably part of the sulfation pathway (Giordano and Prioretti, 2014) and possesses a functional APK domain fused at the ATPS N-terminus (Patron et al., 2008; Figure 3C). The cytosolic isoform of the diatoms and haptophytes is also characterized by a fusion with a pyrophosphatase at the C-terminus of the ATPS domain (Bradley et al., 2009), a similar domain configuration to that observed in oomycetes of the genus *Phytophthora* (Bradley et al., 2009). Although biochemical and kinetic data are not yet available for this enzyme, it has been hypothesized that the pyrophosphatase removes the PPi produced as a by-product of APS synthesis, thereby making the forward ATPS reaction irreversible. For what it is known, the only diatom that appears to constitute an exception to this pattern of isoform localization is *Thalassiosira pseudonana*: in this species, based on the presence/absence of a plastid transit peptide and sequence analysis, it was suggested that the isoform with the sole ATPS domain is located in the cytosol, whereas the APK-ATPS-pyrophosphatase isoform is in the chloroplast (Patron et al., 2008; Bromke et al., 2013). This localization is however to be taken with care, since the sequence was inferred from a raw contig at a time when few protein models were available for *T. pseudonana* genome (N. J. Patron, personal communication); even now, currently available softwares for the prediction of signal/transit peptides are not optimized for secondary endosymbiotic organisms and we were unable to unambiguously determine the nature of this transit peptide. We therefore believe that further and more thorough analyses are required to verify the location of ATPS in *T. pseudonana* and other related organisms.

A peculiar ATPS isoform was found in the dinoflagellates *Heterocapsa triquetra* (Patron et al., 2008), *Amphidinium klebsii* (Giordano and Prioretti, 2014) and *Amphidinium carterae*, in the photosynthetic apicomplexan *Chromera velia* (notice that Apicomplexa group into the superphylum Alveolata together with dinoflagellates) and in the filasterean snail symbiont *Capsaspora owczarzaki* (Suga et al., 2013; it is worthwhile mentioning that the filasterians are among the closest unicellular relative of metazoan). These organisms possess an APR domain fused to the ATPS domain at the N-terminus (Figure 3E). Although it is tempting to conclude that such an arrangement facilitates APS reduction during sulfate assimilation, no data are available on the activity and kinetic of these enzymes and the attribution of any function to this isoform is premature. The similarity between the enzyme of the Filasterea, a group rather closely related to metazoa, and that of alveolates makes the explanation of the origin of ATPS in these organisms difficult. The species of the genus *Amphidinium* also possess an ATPS sequence with the sole ATPS domain (Giordano and Prioretti, 2014). The sequences of *A. klebsii*, *A. carterae*, *C. velia*, and that of the green alga *Tetraselmis suecica*, which are not yet available in public databases, are shown in Table S1 (supplementary materials).

The high diversity of ATPS isoforms may reflect long and independent evolution of lineages as well as adaptation to specific habitats.

## ATPS phylogeny

**Figure 4:**
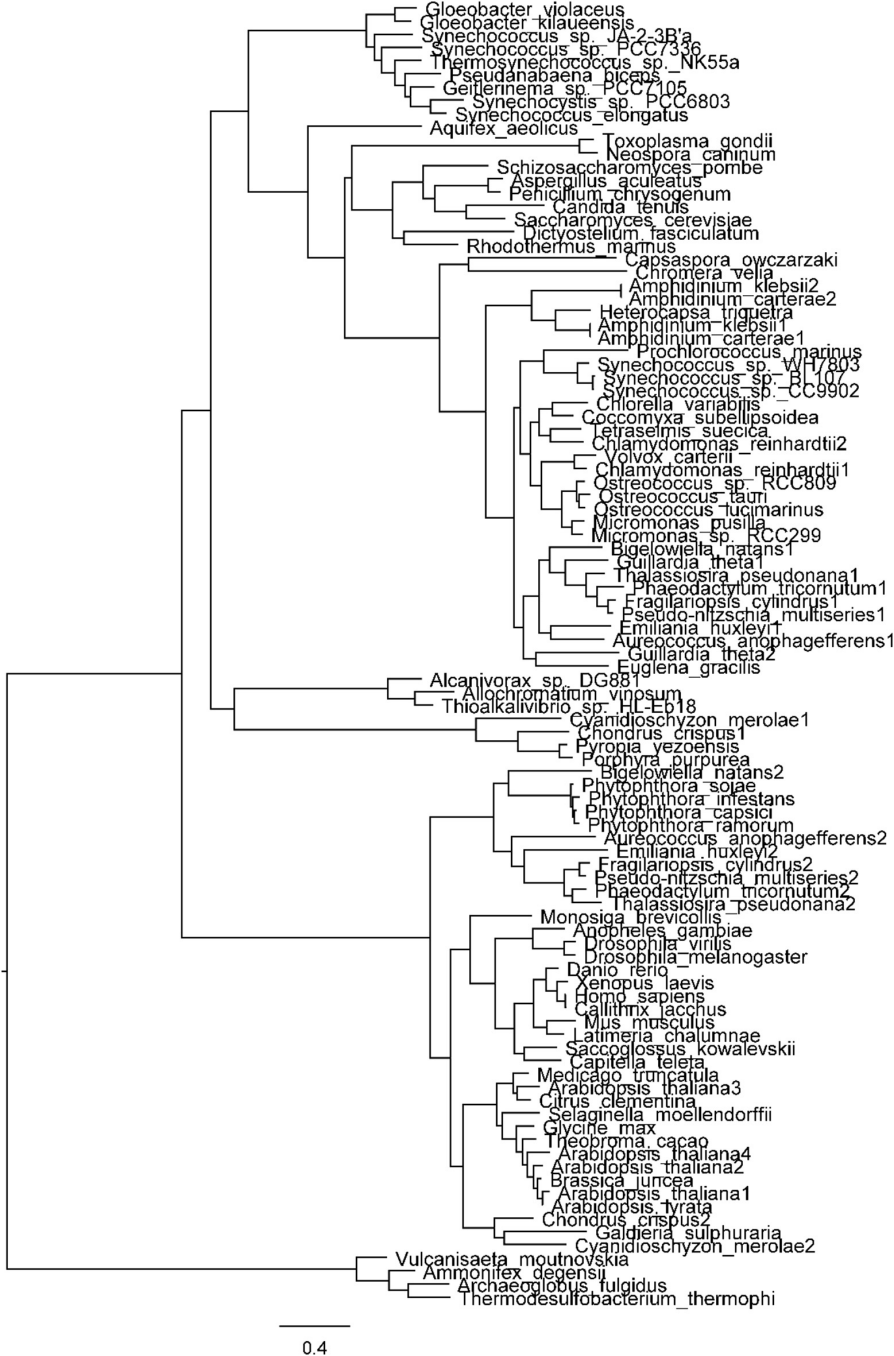
**Phylogenetic tree of ATP sulfurylase**. All protein sequences, except those of *Tetraselmis suecica*, *Amphidinium klebsii*, *Amphidinium carterae*, *Heterocapsa triquetra*, and *Chromera velia* were obtained from either the NCBI protein database (http://www.ncbi.nlm.nih.gov/protein/), using the BLASTp (protein—Basic Local Alignment Search Tool) algorithm (http://blast.ncbi.nlm.nih.gov/Blast.cgi?PROGRAM=blastp&PAGE_TYPE=BlastSearch&LINK_LOC=blasthome), or the JGI (Joint Genome Institute) Genome Portal (http://genome.jgi.doe.gov/); *H. triquetra*, *A. carterae*, and *C. velia* ATPS sequences were kindly provided, respectively, by Stanislav Kopriva (University of Cologne), Charles F. Delwiche (University of Maryland) and Miroslav Obornik (Institute of Microbiology, Czech Academy of Sciences). *T. suecica* and *A. klebsii* sequences were determined by the authors (M.G.) in collaboration with Charles F. Delwiche. The sequences were then aligned using the software MUSCLE (MUltiple Sequence Comparison by Log-Expectation, http://www.ebi.ac.uk/Tools/msa/muscle/). The phylogenetic tree was finally constructed using the software SeaView (version 4, http://pbil.univ-lyon1.fr/software/seaview3.html). The aligned sequences were first modified using the Gblocks function to eliminate all the gaps and N- and C-termini in order to make the sequences comparable. A 10 bootstraps maximum-likelihood phylogenetic tree was then created using the PhyML program. The tree was finally edited with the software FigTree 1.4.0 (http://tree.bio.ed.ac.uk/software/figtree/).

The distribution of the different taxa in the ATPS tree (Patron et al., 2008; Figure 4) complicates the reconstruction of the phylogeny of this gene. It is noteworthy that the base of the tree is occupied by eukaryotic sequences. Patron et al. (2008) suggest that this may, at least to some extent, be the consequence of extensive lateral gene transfer. Interestingly, the ATPS of green algae cluster with that of the cyanobacteria and with the “red lineage” plastidial isoform. It is instead rather distant from the plant enzyme, making a plastidial origin of all extant assimilatory ATPS unlikely (Patron et al., 2008). Also the fungal ATPS-APK clusters with the bacterial enzyme and with the plastidial isoform of eukaryotic algae. On the other hand, the ATPS domains of the APK-ATPS of metazoa and algae with secondary red plastids have high sequence similarity (about 40–45%) with the ATPS of vascular plants, which does not possess the APK domain, but not with the ATPS-APK of fungi. The above ensemble of information is suggestive of the fact that the fused enzymes are derived from evolutionary events that occurred after the appearance of ATPS, whose phylogeny and evolutionary trajectories remain to be elucidated.

## Regulation of ATPS expression and activity

Although sulfate reduction catalyzed by APR is usually considered the most regulated step of S assimilation (Vauclare et al., 2002), there are hints of regulatory processes operating on ATPS activity as well (Giordano and Raven, 2014). The information of ATPS regulation is mostly limited to vascular plants (Leustek and Saito, 1999; Brunold, 2000; Koprivova et al., 2013), which, unfortunately, does not represent the full diversity of photosynthetic organisms.

In vascular plants and algae, ATPS activity is modulated in response to oxidative stress. For instance, an increase of ATPS activity was observed in roots of *Brassica napus* (Lappartient and Touraine, 1997) and *A. thaliana* subject to S deprivation (Lappartient et al., 1999). In *B. napus*, ATPS activity was inhibited by glutathione (Lappartient and Touraine, 1996) and H_2_O_2_ (Lappartient and Touraine, 1997). Also in the diatom *Phaeodactylum tricornutum* ATPS activity was very sensitive to H_2_O_2_ (Rosenwasser et al., 2014). The ATPS activity of the aquatic plants *Lemna gibba* and *Salvinia minima* exposed to arsenic greatly increased, together with the activities of γ-glutamylcysteine synthetase, glutathione S-transferase and glutathione reductase (Leao et al., 2014). An increase in the abundance of ATPS mRNA was observed in *Brassica juncea* roots exposed to Cd, but not in the presence of other heavy metals (Lee and Leustek, 1999). These data suggest that sulfur assimilation, on which ultimately glutathione and phytochelatins synthesis depends, is modulated at its beginning (i.e., at the sulfate activation step catalyzed by ATPS) when cells/plants are confronted with heavy metal and oxidative stress (typically the latter leading to the former type of stress).

In algae, ATPS gene expression (Yildiz et al., 1996; Zhang et al., 2004) and enzyme activity (Giordano et al., 2000; Prioretti and Giordano, unpublished) can be either down-regulated or up-regulated in response to sulfate availability. The transcription of both the genes *ATS1* and -*2* of *C. reinhardtii* is strongly up-regulated by S deprivation and their expression is under the control of the *SAC1* gene, which is responsible for the acclimation to S-limited conditions (Yildiz et al., 1996; Zhang et al., 2004). On the contrary, in the haptophyte *Emiliania huxleyi*, *ATPS1* expression is not affected by S limitation (Bochenek et al., 2013). In *Dunaliella salina*, ATPS activity is up-regulated in response to severe S-limitation leading to major metabolic adjustment concerning also N and C metabolism (Giordano et al., 2000). In the prasinophyte *T. suecica*, the diatom *T. pseudonana* and the marine cyanobacterium *Synechococcus* sp. strain WH7803 ATPS activity increases in response to S limitation. In the case of the dinoflagellate *A. klebsii*, ATPS activity is strongly down-regulated in response to S limitation (Prioretti and Giordano, unpublished).

Changes in the availability of other nutrients or possibly in elemental stoichiometry also appear to influence the expression and activity of ATPS. For example, a decrease of ATPS activity was detected in response to nitrate starvation (causing an increase in the S:N ratio) in *Lemna minor* (Brunold and Suter, 1984). In the haptophyte *Isochrysis galbana*, on the contrary, up-regulation of the ATPS gene expression was detected after nitrogen depletion (Song et al., 2013). Recently, an experiment carried out in order to test the effect of acid rains on plant sulfur metabolism revealed that ATPS gene expression, as the expression of most of the other genes of sulfur metabolism, was up-regulated after a treatment with a solution of 5:1 sulfate and nitrate (Liu et al., 2014). ATPS gene expression also responds to temperature: in *Glycine max* seeds, it was up-regulated in response to low temperature. This response appeared to be mediated by glutathione (Phartiyal et al., 2006). All these findings, together, demonstrate that ATPS is involved in a variety of cellular functions and is central to a number of metabolic adjustments possibly associated with the requirement for compositional homeostasis (Montechiaro and Giordano, 2010; Giordano, 2013).

The mechanisms of ATPS regulation, however are not fully understood. Recent findings indicate that ATPS gene expression in *A. thaliana* is, at least in part, controlled post-transcriptionally by the miR395 family of micro-RNAs (miRNAs, non-coding short RNAs), whose production is induced by sulfate deprivation (Jones-Rhoades and Bartel, 2004; Allen et al., 2005; Kawashima et al., 2011). miR395 is regulated by SLIM1 (Kawashima et al., 2011), a transcription factor responsible of the regulation of sulfate transporters *SULTR1;1*, *−1;2*, *−3;4*, and *−4;2* and of *ATPS4* during S limitation (Maruyama-Nakashita et al., 2006). The target of miR395 are *ATPS1*, −*3* and −*4* and *SULTR2;1* expression. *ATPS1* and *SULTR2;1* mRNA are post-transcriptionally degraded in the phloem companion cells where miR395 is expressed. However, the way these micro-RNAs perform their functions and their effects on *ATPS3* and *ATPS4* expression is still unclear.

Recent findings showed that miR395 expression is also sensitive to various kinds of environmental stresses, such as salt and drought stress (Wang et al., 2013). Oxidative stress mediated by copper and arsenate ions also induces miR395, whereas the addition of GSH suppresses this induction (Jagadeeswaran et al., 2014). These findings suggest that miR395 expression might be mediated also by redox signaling. The lack of a conserved miR395 family in *C. reinhardtii* suggests that green algae miRNAs may have a different mode of response to sulfur-deprivation (if any) than in higher plants (Shu and Hu, 2012).

Evidence for transcriptional regulation of *ATPS* expression was found in *A. thaliana* by Yatusevich et al. (2010). These authors reported that *ATPS1* and *ATPS3* expression was directly regulated by members of the R2R3-MYB transcription factors family, which are responsible of the synthesis of glucosinolates (GSs). GSs are a group of sulfated metabolites the synthesis of which involves catalysis by sulfotransferases (SOTs). These enzymes use 3′-phoshoadenosine-5′-phosphosulfate (PAPS) as a source of sulfate for the sulfation of amino acid-derived thioglucosides (Sonderby et al., 2010). Their synthesis is also regulated by SLIM1 when S is limiting (Maruyama-Nakashita et al., 2006). A specific control of GSs synthesis and of the expression of *ATPS1* and *ATPS3* by the MYB transcription factors was found: *ATPS1* expression is mainly regulated by MYB transcription factors which control the synthesis of aliphatic GSs, whereas *ATPS3* expression is associated with MYB transcription factors which control the synthesis of indolic GSs (Yatusevich et al., 2010). No relationships between MYB transcription factors and *ATPS2* and *ATPS4* expression has been detected until now. The above information point to a rather complex regulation of ATPS, although the exact mode of action is not always clear. The case of *A. thaliana* suggests that different ATPS isoforms may be differently regulated and may play different metabolic roles (Kopriva et al., 2009).

## Do number and position of cysteine residues in ATPS sequences have significance for redox regulation?

ATPS enzymatic activity might be sensitive to redox regulation in some photosynthetic organisms and it was reported to be a target for thioredoxins (TRXs; see Buchanan and Balmer, 2005; Balsera et al., 2014 for specific reviews on TRXs) in both plants (Dixon et al., 2005; Marchand et al., 2006) and cyanobacteria (Lindahl and Florencio, 2003). The response of ATPS activity to oxidative stress mentioned above (Lappartient and Touraine, 1996, 1997; Rosenwasser et al., 2014) point to that direction.

**Figure 5:**
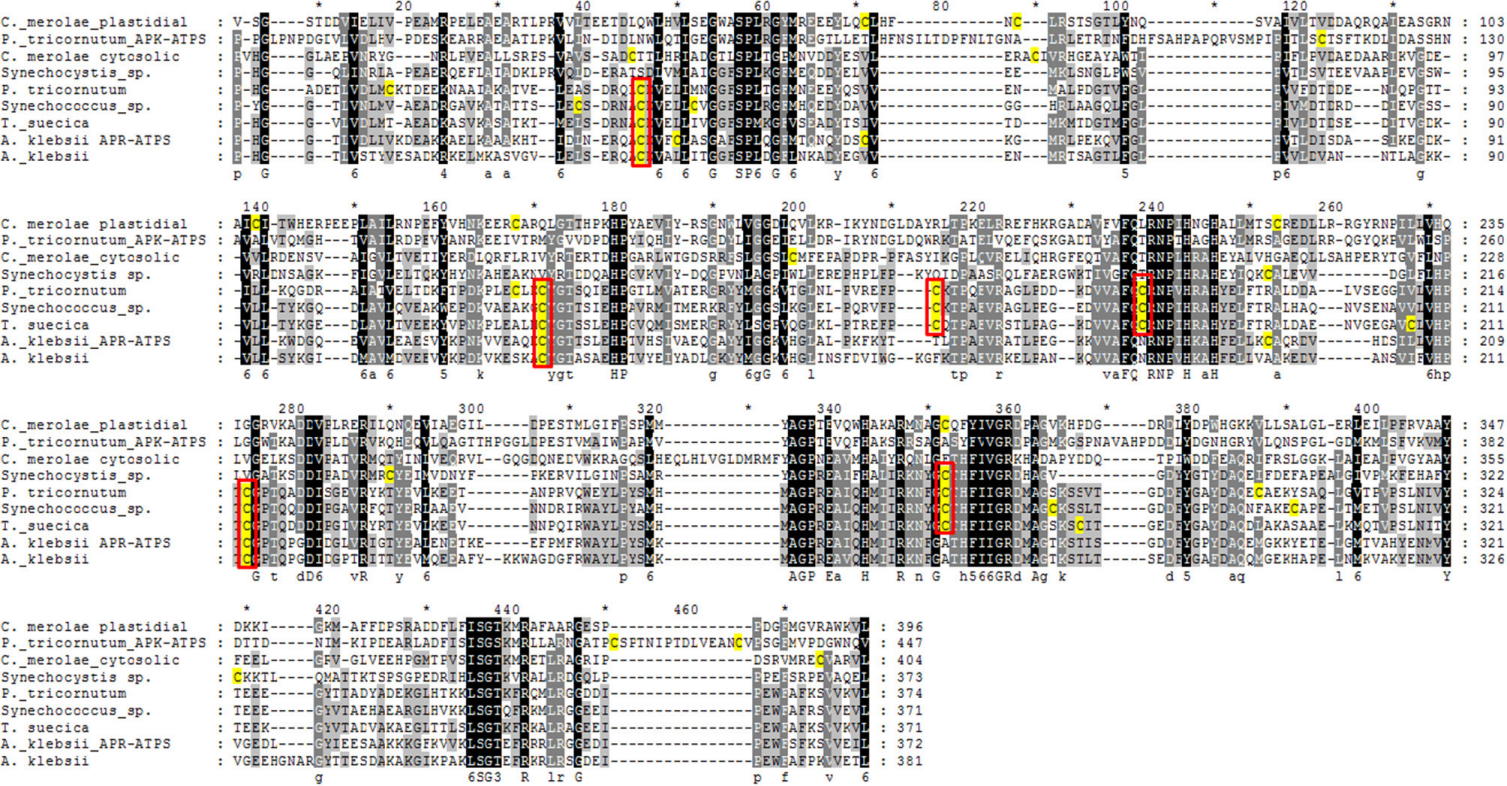
**Multiple sequence alignment of the sole ATP sulfurylase domain from different algal species showing cysteine residues (highlighted in yellow) and their conservation among species**. A representative for each group of algal ATPS (see Figure 3) was chosen: *Tetraselmis suecica* was selected for green algae, *Phaeodactylum tricornutum* for diatoms and haptophytes (both the enzyme with the sole ATPS function and the ATPS domain in the APK-ATPS enzymes are shown), *Cyanidioschyzon merolae* for red algae (one plastidial and one cytosolic ATPS are present in this species, both constituted by the sole ATPS domain), *Amphidinium klebsii* for dinoflagellates (both the monofunctional ATPS and the ATPS domain of the APR-ATPS enzyme are shown), *Synechococcus* sp. strain WH7803 for marine cyanobacteria and *Synechocystis* sp. strain PCC6803 for freshwater cyanobacteria. The cysteine residues included in a red rectangle are present, and their position is conserved, in all the ATPS sequences belonging to the same group (as described in Figure 3). Alignments were performed using MUSCLE (http://www.ebi.ac.uk/Tools/msa/muscle/) and results exported and edited with GenDoc (http://www.nrbsc.org/gfx/genedoc/).

Cysteine residues are among the most likely targets for redox regulation mechanisms (Couturier et al., 2013). Consequently, we studied the ATPS sequences of photosynthetic organisms to verify if the response to reducing and oxidizing agents was correlated with the number and position of cysteine residues. This study revealed that algal ATPS proteins contain an unexpected high number of cysteine residues with respect to the ATPS from plant and other organisms, and many of the algal cysteine residues are conserved (Table 1). Most algal ATPS proteins (except those of red algae—see below) contain 5–10 cysteine residues in their sequences, in contrast to the ATPS of vascular plants and fungi, which at the most contains two cysteine residues. The additional residues in algal sequences are often in positions different from those of the cysteine residues of other organisms. Among algae, five main groups of ATPS proteins can be identified with respect to the number and location of the cysteine residues:

- The ATPS of freshwater cyanobacteria and of marine cyanobacteria that do not belong to the genera *Synechococcus* and *Prochlorococcus*; these enzymes contain 4 conserved cysteine residues.
- The plastidial ATPS of Chlorophyta, Cryptophyta, Haptophyta and Heterokontophyta (notice the exception of *T. pseudonana* cytosolic ATPS mentioned above) and the enzyme of marine cyanobacteria of the genera *Synechococcus* and *Prochlorococcus* (possibly the most abundant marine cyanobacteria; Flombaum et al., 2013). These ATPS proteins constitute a consistent phylogenetic group (Patron et al., 2008) and contain 7–10 cysteine residues, 5 of which are highly conserved (Figure 5), although at positions different from those of the ATPS of the group A. The ATPS sequence from *E. gracilis* can be included in this group since it has 3 conserved cysteine residues at the same position as some of those of the group B.
- ATPS from red algae; 1–7 cysteine residues are present in these sequences and, although some cysteine residues are conserved among some of the species inside this group, their position varies and no common pattern can be identified.
- The cytosolic bi-functional APK-ATPS enzymes of algae with red secondary plastid; in this case, 5–8 cysteine residues are present in the ATPS domain of the protein, two of which are conserved, but at positions different from those of the cysteine residues of the ATPS of the other groups.
- Dinoflagellate ATPS; these proteins are different from all those mentioned above. At this stage, sequence information is available only for three species *H. triquetra* (Patron et al., 2008), *A. klebsii* (Prioretti and Giordano, unpublished) and *A. carterae*. It may therefore be unwise to draw general conclusion based on these species only. All the three species possess an enzyme with fused APR and ATPS domains. These APR-ATPS proteins contain 7 cysteine residues in the ATPS domain, one of which is at the same position as one of the cysteine residues of the enzymes from group A and two have the same position as two of the cysteine residues of group B ATPS. In *A. klebsii*, also a protein with the sole ATPS domain is present. It contains 6 cysteine residues, two of which are at the same positions as the cysteine residues of the bi-functional APR-ATPS enzyme (Figure 5).

**Table 1:** **ATP sulfurylase (ATPS) of algae and their cysteine content**.

**Phylum**	**Species**	**Environ**.	**Number and type of genes**	**Localization**	**Total number of Cys**	**Number of conserved Cys**
Cyanobacteria	*Acaryochloris* sp.	M	1 ATPS	–	4	4 A
	*Anabaena* sp.	F	1 ATPS	–	4	4 A
	*Arthrospira platensis*	F	1 ATPS	–	4	4 A
	*Crocosphaera* sp.	M	1 ATPS	–	4	4 A
	*Cyanobacterium* sp.	M	1 ATPS	–	4	4 A
	*Cyanothece* sp.	M	1 ATPS	–	4	4 A
	*Cylindrospermopsis* sp.	F	1 ATPS	–	5	4 A
	*Fischerella* sp.	F	1 ATPS	–	4	4 A
	*Gloeobacter* sp.	F	1 ATPS	–	4	4 A
	*Lyngbia* sp.	M	1 ATPS	–	4	4 A
	*Microcoleus vaginatus*	F	1 ATPS	–	4	4 A
	*Microcystis aeruginosa*	F	1 ATPS	–	4	4 A
	*Nodularia* sp.	M	1 ATPS	–	4	4 A
	*Nostoc* sp.	F	1 ATPS	–	4	4 A
	*Oscillatoria* sp.	F	1 ATPS	–	4	4 A
	*Prochlorococcus marinus*	M	1 ATPS	–	9-11	5 B
	*Raphidiopsis* sp.	F	1 ATPS	–	4	4 A
	*Spirulina subsalsa*	M	1 ATPS	–	4	4 A
	*Synechococcus elongatus*	F	1 ATPS	–	4	4 A
	*Synechococcus* sp. CB0101	M	1 ATPS	–	8	5 B
	*Synechococcus* sp. CC9902	M	1 ATPS	–	10	5 B
	*Synechococcus* sp. JA-3-3Ab	F	1 ATPS	–	5	4 A
	*Synechococcus* sp. JA-2-3B'a	F	1 ATPS	–	6	4 A
	*Synechococcus* sp. RCC307	M	1 ATPS	–	8	5 B
	*Synechococcus* sp. RS9916	M	1 ATPS	–	9	5 B
	*Synechococcus* sp. WH7803	M	1 ATPS	–	10	5 B
	*Synechococcus* sp. WH8102	M	1 ATPS	–	9	5 B
	*Synechocystis* sp. PCC6803	F	1 ATPS	–	4	4 A
	*Thermosynechococcus* sp.	F	1 ATPS	–	6	4 A
	*Trichodesmium* sp.	M	1 ATPS	–	4	4 A
Chlorophyta	*Chlamydomonas reinhardtii*	F	1 ATPS	P	9	5 B
	*Chlamydomonas reinhardtii*	F	1 ATPS	P	10	5 B
	*Chlorella variabilis*	F	1 ATPS	P	12	5 B
	*Coccomyxa subellipsoidea*	F	1 ATPS	P	10	5 B
	*Micromonas pusilla*	M	1 ATPS	P	8	5 B
	*Ostreococcus lucimarinus*	M	1 ATPS	P	9	5 B
	*Ostreococcus tauri*	M	1 ATPS	P	8	5 B
	*Tetraselmis suecica*	M	1 ATPS	U	8	5 B
	*Volvox carteri*	F	1 ATPS	P	9	5 B
Euglenozoa	*Euglena gracilis*	F	1 ATPS	M	6	3 B
Rhodophyta	*Chondrus crispus*	M	1 ATPS	U	1	3 (1 B; 2 E)
	*Chondrus crispus*	M	1 ATPS	U	5	0
	*Cyanidioschyzon merolae*	M	1 ATPS	P	7	3 (1 B; 2 E)
	*Cyanidioschyzon merolae*	M	1 ATPS	C	4	0
	*Galdieria sulphuraria*	M	1 ATPS	U	5	3 (1 B; 2 E)
	*Porphyra purpurea*	M	1 ATPS	C	2	1 C
	*Pyropia yezoensis*	M	1 ATPS	C	1	1 C
Chromerida	*Chromera velia*	M	1 ATPS	U	7	2 B
Cryptophyta	*Guillardia theta*	M	1 ATPS	P	9	5 B
Dinophyta	*Amphidinium carterae*	M	1 ATPS	U	6	2 B
	*Amphidinium carterae*	M	1 APR-ATPS	U	7	3 (1 A; 2 B)
	*Amphidinium klebsii*	M	1 ATPS	U	6	2 B
	*Amphidinium klebsii*	M	1 APR-ATPS	U	7	3 (1 A; 2 B)
	*Heterocapsa triquetra*	M	1 APR-ATPS	P	7	3 (1 A; 2 B)
Haptophyta	*Emiliania huxleyi*	M	1 ATPS	P	9	5 B
	*Emiliania huxleyi*	M	1 APK-ATPS	C	5	2 D
Heterokontophyta	*Aureococcus anophagefferens*	M	1 ATPS	U	17	5 B
	*Aureococcus anophagefferens*	M	1 APK-ATPS	U	7	2 D
	*Ectocarpus siliculosus*	M	1 ATPS	P	10	5 B
	*Fragilariopsis cylindrus*	M	1 ATPS	U	8	5 B
	*Fragilariopsis cylindrus*	M	1 APK-ATPS	U	6	2 D
	*Phaeodactylum tricornutum*	M	1 ATPS	P	9	5 B
	*Phaeodactylum tricornutum*	M	1 APK-ATPS	C	8	2 D
	*Pseudo-nitzschia multiseries*	M	1 ATPS	U	7	5 B
	*Pseudo-nitzschia multiseries*	M	1 APK-ATPS	U	5	2 D
	*Thalassiosira pseudonana*	M	1 ATPS	C	8	5 B
	*Thalassiosira pseudonana*	M	1 APK-ATPS	P	7	2 D

*All species for which the ATPS sequence is known are included in the table, except for Cyanobacteria, for which only representative species or strains of the oceanic genera Prochlorococcus and Synechococcus are shown. The column “Environ.” indicates whether the species is marine (M) or freshwater (F). The column “Number and type of genes” indicates how many ATPS are present and of what type (i.e., if the protein contains the sole ATPS domain, if the protein contains an APR domain, etc.). The column “Localization” shows whether the protein is plastidial (P), cytosolic (C), mitochondrial (M) or unknown (U); in the case of prokaryotes, no compartmentation is indicated. The column “Total number of Cys” indicates how many cysteine residues are contained in the ATPS domain of the enzyme. The column “Number of conserved Cys” refers to the number of cysteine residues that are retained at the same position in the sequences; residues in the same position are identified by the same letter (A, B, C, D or E). All sequences, except those of Tetraselmis suecica, Amphidinium klebsii, Amphidinium carterae, Heterocapsa triquetra and Chromera velia were obtained from either the NCBI protein database or the JGI genome database; H. triquetra, A. carterae, and C. velia ATPS sequences were kindly provided, respectively, by Stanislav Kopriva (University of Cologne), Charles F. Delwiche (University of Maryland) and Miroslav Obornik (Institute of Microbiology, Czech Academy of Sciences). T. suecica and A. klebsii sequences were produced by the authors in collaboration with Charles F. Delwiche*.

Preliminary experiments conducted in MG laboratory showed that the number and location of the cysteines residues appear to be related to the sensitivity of the enzyme activity to thiol reducing and oxidizing agents, with the enzyme of group B being redox regulated, as opposite to those of group A and E. Further experiments are being conducted to check if and to what extent this information has general significance.

## Evolutionary trajectories of algal ATPS

The above grouping of alga ATPS and their phylogenetic relationships show one rather surprising fact: unlike the majority of cyanbacterial genes, which are phylogenetically closer within cyanobacteria than with respect to all other phyla (Zhaxybayeva et al., 2006), ATPS proteins of the *Synechococcus* and *Prochlorococcus* genera are closer to those of the eukaryotic algae than to those of all other cyanobacteria. Speculatively, the difference between the two groups of cyanobacteria may reflect the chemistry of the environments in which aquatic photosynthetic organisms live: sulfate concentration usually ranges between 10 and 800 μmol L^−1^ in freshwaters, 700–800 μmol L^−1^ can be found in eutrophic lakes, a maximum of 1 mmol L^−1^ is reached in brackish waters (Holmer and Storkholm, 2001). In the ocean, sulfate concentrations underwent a monotonic increase over time, until they reached the present maximum of 28–30 mmol L^−1^ in the Mesozoic era (Canfield, 2004; Ratti et al., 2011). The larger number of cysteines in the dominating oceanic cyanobacteria (Flombaum et al., 2013) may thus be associated to the higher availability of sulfate in extant seawater. On the other hand, the fact that most of eukaryotic algae, regardless of whether they inhabit freshwater or marine waters, have the same type of ATPS indicates that the number of cysteine residues is not a decisive feature for life in the oceans or it is a frozen accident of ATPS evolutionary trajectories in eukaryotes. Whether eukaryotic algae inherited their ATPS from marine cyanobacteria (or from a common ancestor which also gave rise to the ATPS of marine cyanobacteria of the *Synechococcus* and *Prochlorococcus* genera), or the extant distribution of ATPS isoforms is the consequence of lateral gene transfers between eukaryotic algae and marine cyanobacteria cannot be determined with the information at hand. Certainly, the existence of multiple and different ATPS isoforms in some algae (rhodophytes, diatoms and dinoflagellates, just to mention a few) is suggestive of a multiple and complex origin for this enzyme.

### Conflict of interest statement

The authors declare that the research was conducted in the absence of any commercial or financial relationships that could be construed as a potential conflict of interest.
