# Supplementary material for: Diversity and regulation of ATP sulfurylase in photosynthetic organisms
Source: Front Plant Sci. 2014 Nov 5;5:597. doi: 10.3389/fpls.2014.00597 (PMC4220642; doi:10.3389/fpls.2014.00597)
Supplement: Supplementary file 1 [file Table1.PDF]

Table S1. The ATPS enzymes whose sequences are still not available in public databases are shown in this table. The column “Type of enzyme” indicates if the enzyme is a mono-functional ATPS or a bi-functional APR-ATPS. For the bi-functional enzymes, the amino acid sequence of the domains corresponding to the APR and the ATPS are separated by a dash.

Phylum	Species	Type of enzyme	Amino acid sequence	Source
Chlorophyta	*Tetraselmis suecica*	ATPS	MAFAVSAQATAAVAATQASASAGRRTAAARAVAAPTPVRSLRSFAARQQSLTAAGRRAASIRASAAVRA<br>AAVEAGLQEPHGGVLVDLMTAEADKASVKASATKTMELSDRNACDVELLIVGGFSPMKGFVSEADYTSI<br>VTDMKMTDGTMFGLPIVLDTDSEDITVGDKVLLTYKGEDLAVLTVEEKYVPNKPLEALNCYGTSSLEHPG<br>VQMISMERGRYYLSGPVQGLKLPTREFPCQTPAEVRSTLPAGKDVVAFQCRNPIHRAHYELFTRALDAEN<br>VGEGAVCLVHPTCGPTQDDDIPGIVRYRTYEVLKEEVNNPQIRWAYLPYSMHMAGPREAIQHMIIRKNYG<br>CTHFIIGRDMAGSKSCITGEDFYGAYDAQDLAKASAAELKMQTVPSLNITYTEEKGYVTADVAKAEGLTT<br>LSLSGTKFRKALRAGEEIPEWFAFKSVVKVLREA	Charles F. Delwiche (University of Maryland)<br>Mario Giordano (Università Politecnica delle Marche)
Chromerida	*Chromera velia*	APR-ATPS	MSLSCVSRTFLSRAAAPRATSLLQGRLHGAASASRFPLNGAFASSVVRPMSTWTREDWGAIPKDSAVLKP<br>VSSDTEHRLKQLVKEFQELSKERLEGKNSVIITAWLVTDNVIADLFKRFTNARPELVAVDTLHIFPETRTCA<br>KEMDSIGLKTLWYKPKGCETLEDFQKKFGHYNALKDDDFDYSSKVEPLQQAMNELKSKSAVVITGRRND<br>QGNARTSIPTWEEERNTLNPLVDWTWEDVTAYATMYNVPVNSLHKRVLLAENEISPLTRDAHKDFLEFN<br>LDRPYWEYNEAALEKMFGGKHWYVWKSFGDFHTSLPVKIHESERTGRFVKRVATECGIHTRV - SNTKK<br>APHGGSKLVNLLPSAFRPAPDVSQCKHTISLTERQLCDLELLINGGYTPLNGFMTEAEYDHSLLHMRLPEG<br>AVWGLPVVLDIGAEMASKVQEGDKVLLRADGFGGDLAVMTVTSKWNPDKKAEALHTCSTDAADHPYV<br>GYMYNQMGETYIGGPIEGIRLPERPWVRRVSTPSETREKLKKAEEVVAFQCRNPLHKAHVAMFTDTAAA<br>KIPTVVHPAIGPSKDDDFDASVRIPTYETLNRTLADQMISFEYFPYHMRLAGPREAVQHMICRKNYGFTQM<br>IIGRDHAGCKNAAGEDFYGPYDAQELASSVEAEIGITTVPFKAYVFHADSSSYVTKEDAKERGLKTVNISG<br>TEFRRRITAGEAVPDWYAFPDVVQLLQASYNVDRTAKAEEVPKLAEGSERAAVLMRGGDASAQEALAKI<br>SNAKTHILFFHPEKEAGASVEFDVFGPKGQKLRTCGSLEELEAFLQGI	Miroslav Oborník (Institute of Microbiology, Czech Academy of Sciences)<br>Arnab Pain (King Abdulah University of Science and Technology, Tuwal, Saudi Arabia)
Dinophyta	*Amphidinium carterae*	ATPS	MMKVAILCALLALLPGSAANAEEGTCDAKTGVCTADAGKPHGGTLVSTYVESADKRKELMKASVGVLE<br>LSERQACDVALLITGGFSPLDGFLNKADYEGVVENMRTSAGTLFGLPVVLDVANNTLAGKKVLLSYKGID<br>MAVMDVEEVYKPDKVKESKACYGTASAEHPTVYEIYADLGKYYMGGKVHGLINSFDVIWGKGFKTPAE<br>VRKELPANKQVVAFQNRNPVHKAHFELLVAAKEDVANSVIFVHPTCGPTQPGDIDGPTRITTYEVMQEEA<br>FYKKWAGDGFRWAYLPYSMKMAGPREAVQHMIIRKNFGATHFIIGRDMAGTKSTLTSEDFYGAFDAQQ<br>MGEKHAPELNMKVAKYENMVYVGEEHGNARGYTTESDAKAKGIKPAKLSGTEFRKRLRSGDEIPEWFA<br>FPKVVETLRAGGDKIFL	Charles F. Delwiche (University of Maryland)

	APR-ATPS	MFVLSVASPLLKRTVGAAPVRRLIQVRSFAALSDLEEKFKADVKKHLPADGKGVAFATAWLATDNVFVA<br>LLHKHFPDVLKGMTLVAIDTMHLFPDALVCAKEVQDKYSKAALVKVPKGVTTREEFEATYGDCESLDTA<br>DFDFVSKVEPFQRALAECKKEILITGRRMDQAAQRIDLDIWEEGKKTMNPMAEWSFSDITSYADANGVPV<br>NKGHNLAFRCQAEIEATSRHLPDLPWTKVDMGKPFWQCTLDELKGGSPIVYVYKSFGDTHTTVPVMPHE<br>TERAGRFVRQAKTECGIHTR - TTSEGAPHGGTLVDLLVKDEAKKAELKAAAKHTIDLNERQACDVFCLA<br>SGAFSPLQGFMTQNQYDSCVKGMRLPEKQVFGLPVTLDISDASIKEGDKVLLKWDGQEVAVLEAESVYK<br>PNKVVEAQECYGTTSLEHPTVHSIVAEQGAYYIGGKVHGLALPKFKYTTLTPAEVRATLPEGKKVVAFQN<br>RNPIHKAHFELLKCAQRDVHDSILLVHPTCGPTQPGDIDGLVRIGTYEALENETKEEFPMFRWAYLPYSMK<br>MAGPREAIQHMIIRKNFGATHFIIGRDMAGTKSTISGDDFYGPYDAQEMGKKYETELGMTVAHYENMVY<br>VGEDLGYIEESAAKKKGFKVVKLSGTEFRRRLRGGEDIPEWFSFKSVVEILRKAGDKAFVA	Charles F. Delwiche (University of Maryland)
Amphidinium klebsii	ATPS	MMKVAILCALLALLPGSAANAEEGTCDAKTGVCTADAGKPHGGTLVSTYVESADKRKELMKASVGVLE<br>LSERQACDVALLITGGFSPLDGFLNKADYEGVVENMRTSAGTLFGLPVVLDVANNTLAGKKVLLSYKGID<br>MAVMDVEEVYKPDKVKESKACYGTASAEHPTVYEIYADLGKYYMGGKVHGLINSFDVIWGKGFKTPAE<br>VRKELPANKQVVAFQNRNPVHKAHFELLVAAKEDVANSVIFVHPTCGPTQPGDIDGPTRITTYEVMQEEA<br>FYKKWAGDGFRWAYLPYSMKMAGPREAVQHMIIRKNFGATHFIIGRDMAGTKSTLTSEDFYGAFDAQQ<br>MGEKHAPELNMKVAKYENMVYVGEEHGNARGYTTESDAKAKGIKPAKLSGTEFRKRLRSGDEIPEWFA<br>FPKVVETLRAGGDKIFL	Charles F. Delwiche (University of Maryland)<br><br>Mario Giordano (Università Politecnica delle Marche)
	APR-ATPS	MFVLSVASPLLKRTVGAAPVRRLIQVRSFAALSDLEEKFKADVKKHLPADGKGVAFATAWLATDNVFVA<br>LLHKHFPDVLKGMTLVAIDTMHLFPDALVCAKEVQDKYSKAALVKVPKGVTTREEFEATYGDCESLDTA<br>DFDFVSKVEPFQRALAECKKEILITGRRMDQAAQRIDLDIWEEGKKTMNPMAEWSFSDITSYADANGVPV<br>NKGHNLAFRCQAEIEATSRHLPDLPWTKVDMGKPFWQCTLDELKGGSPIVYVYKSFGDTHTTVPVMPHE<br>TERAGR - FVRQAKTECGIHTRTTSEGAPHGGTLVDLLVKDEAKKAELKAAAKHTIDLNERQACDVFCLA<br>SGAFSPLQGFMTQNQYDSCVKGMRLPEKQVFGLPVTLDISDASIKEGDKVLLKWDGQEVAVLEAESVYK<br>PNKVVEAQECYGTTSLEHPTVHSIVAEQGAYYIGGKVHGLALPKFKYTTLTPAEVRATLPEGKKVVAFQN<br>RNPIHKAHFELLKCAQRDVHDSILLVHPTCGPTQPGDIDGLVRIGTYEALENETKEEFPMFRWAYLPYSMK<br>MAGPREAIQHMIIRKNFGATHFIIGRDMAGTKSTISGDDFYGPYDAQEMGKKYETELGMTVAHYENMVY<br>VGEDLGYIEESAAKKKGFKVVKLSGTEFRRRLRGGEDIPEWFSFKSVVEILRKAGDKAFVA	Charles F. Delwiche (University of Maryland)<br><br>Mario Giordano (Università Politecnica delle Marche)
